# Disgust Sensitivity and the Neurophysiology of Left-Right Political Orientations

**DOI:** 10.1371/journal.pone.0025552

**Published:** 2011-10-19

**Authors:** Kevin B. Smith, Douglas Oxley, Matthew V. Hibbing, John R. Alford, John R. Hibbing

**Affiliations:** 1 Department of Political Science, University of Nebraska-Lincoln, Lincoln, Nebraska, United States of America; 2 Department of Political Science, Texas A&M University, College Station, Texas, United States of America; 3 School of Social Sciences, Humanities and Arts, University of California Merced, Merced, California, United States of America; 4 Department of Political Science, Rice University, Houston, Texas, United States of America; University of Bologna, Italy

## Abstract

Disgust has been described as the most primitive and central of emotions. Thus, it is not surprising that it shapes behaviors in a variety of organisms and in a variety of contexts—including homo sapien politics. People who believe they would be bothered by a range of hypothetical disgusting situations display an increased likelihood of displaying right-of-center rather than left-of-center political orientations. Given its primal nature and essential value in avoiding pathogens disgust likely has an effect even without registering in conscious beliefs. In this article, we demonstrate that individuals with marked involuntary physiological responses to disgusting images, such as of a man eating a large mouthful of writhing worms, are more likely to self-identify as conservative and, especially, to oppose gay marriage than are individuals with more muted physiological responses to the same images. This relationship holds even when controlling for the degree to which respondents believe themselves to be disgust sensitive and suggests that people's physiological predispositions help to shape their political orientations.

## Introduction

Associating physiological variation with political issue preferences or political ideology may seem far-fetched but is consistent with longstanding findings from behavior genetics indicating that political orientations exhibit evidence of substantial genetically heritability [1]–[6]. Since a direct connection between genes and variables as context dependent and evolutionarily recent as political attitudes is unlikely, if the goal is to understand biological influences on political attitudes and behavior it makes sense to investigate the connection between physiological systems and political orientations. Certain features of human central and autonomic nervous systems, for example, are clearly encoded in DNA and are also likely to influence perceptions and reactions to the environment, including the political environment. Recent work has begun to examine these sorts of links by correlating variations in distinct neurophysiological patterns with political orientations. The work of Amodio et al. reported a tendency of individuals falling on the right of the ideological spectrum to exhibit a relatively structured and persistent cognitive style; those on the left, a more open and ambiguity-tolerant style [7]. Their findings offered evidence of an association between left-right self-identification and both ERN and ERP amplitudes localized in the dorsal anterior cingulate cortex. In a study conducted by Oxley et al., left-right differences on a collection of political issues relevant to societal protection were found to be associated with differences in both skin conductance and orbicularis oculi startle blink (EMG) induced by threatening images and acoustic startle [8].

In this study we continue the process of connecting political attitudes to neurophysiology by investigating whether physiological responses to disgust stimuli correlate with specific political attitudes already known to correlate with self-report measures of disgust sensitivity. We seek to test two specific hypotheses. First, based on previous research connecting self-reported disgust sensitivity to opposition to opposition to gay marriage [9], [10], we hypothesize that physiological responses to disgust stimuli will positively correlate with attitudes towards gay marriage and will do so even when controlling for the effects of self-reported disgust sensitivity. Second, and also based on previous research, we hypothesize that physiological responses to disgust stimuli will have weaker but still noticeable effects on other sex-related attitudes (for example, those pertaining to pre-marital sex, to pornography, or to abortion) but not to most other political issues (for example, those pertaining to economic and to defense policies).

Disgust has been referred to as “the most visceral of all basic emotions” [11] and the lust-disgust axis is often seen as the original building block of all emotions [12]. The role of disgust in the avoidance of disease, one of the primary sources of mortality over the centuries, makes it essential to survival [13]. Numerous connections between disgust responses and social behavior have been identified [14]–[16]. The foundation for hypothesizing a connection between disgust response and political behavior more specifically is anchored the groundbreaking work of Haidt and colleagues [17], [18]. On the basis of numerous large N surveys, Haidt reports that people on the left make judgments primarily on the basis of two “moral foundations:” harm avoidance and a desire for fairness/equity. People on the political right, on the other hand, display similar attention to harm avoidance and fairness but demonstrate additional concerns for purity, in-group/loyalty, and authority/structure. Interestingly, these differences in moral foundations hold up across cultures [18], a finding consistent with the work of Schwartz on cross-cultural similarity in the relationship between political orientations and patterns of values as well as work on the relationship between political orientations and personality traits across cultures [19]–[21]. This nuanced view of differentially weighted decision considerations is the basis for expecting people on the right to be more likely to emphasize purity/disgust as a foundation for moral and political orientations.

Following on these theoretical expectations as well as the empirical work of Haidt and Hersch [9], Inbar, Pizarro, and Bloom tested for the effects of self-reported disgust sensitivity on left-right ideological location and found that, compared to people on the left, those on the right tended to report being more disgust sensitive. By looking at the correlates of ten individual issue positions, they also investigated the possibility that self-reported disgust sensitivity correlated differentially with selected political issue attitudes. Specifying the particular political attitudes that might be affected by disgusting stimuli makes a good deal of sense since amorphous ideologies are likely to be affected by many other predispositions. Their expectation was that “because disgust is specifically associated with perceived violations of purity-related norms important to those on the right, disgust sensitivity should be especially associated with conservative attitudes on issues related to sexual purity” [10]. The appeal of this approach was borne out in the empirical research of both Haidt and Hersch and of Inbar, Pizarro, and Bloom since in each case it was indeed found that self-reported disgust sensitivity had a powerful connection to issues related to sex and especially to homosexuality but not to a wide range of non-sexually-related policy issues [9], [10]. The finding of a strong relationship between self-reported disgust sensitivity and attitudes toward gay marriage comports nicely with conventional wisdom that for some people opposition to homosexual rights derives from a sense that the very thought of homosexual sex is disgusting and raises the question of why some people would find this thought disgusting while many others do not.

Informative as it is, this previous research is not direct evidence of a connection between political orientations and a neurophysiological mechanism. Disgust is undoubtedly physiological but to date research has dealt with it only in the form of survey based self-reports of a mostly hypothetical nature. The common procedure has been to use the 25-item revised disgust sensitivity survey or DS-R (for a full discussion, see the Disgust Scale Homepage at http://people.virginia.edu/~jdh6n/disgustscale.html). Sample items on the DS-R include “Even if hungry, I would not drink a bowl of my favorite soup if it had been stirred by a used but thoroughly washed flyswatter,” and “it would bother me tremendously to touch a dead body.” The DS-R has consistently been found to have three subfactors (core, contamination, and animal reminder) and, since purity concerns logically match with “core and contamination sensitivity” [18], the contamination and core subcomponents of the full 25-item battery (items dealing with excrement, vomit, unwell individuals, and contaminated or dangerous foods) are typically employed to assess the correlates of political orientations. In contrast, items relating to so-called animal reminder disgust (a subfactor that focuses on violations of the bodily envelop) are removed since they have been found to have less of a connection to politics [22].

Self-reports of disgust sensitivity undoubtedly measure something real and important. The DS-R has been validated in numerous studies and contexts, it correlates with other concepts in sensible fashions, has been connected to real behavior, and has been correlated positively with activity in the anterior insula, an area of the brain known to be relevant to disgust [23]. Still, individuals have been known to adjust self-reports to appear more socially acceptable [24] and, dissembling aside, many people are not particularly adept at identifying their likely reactions to hypothetical situations. Moreover, the issue of concern here is the possible relevance of forces outside the realm of conscious thought and therefore outside the realm of self-reports. Thus, we turn instead to standard physiological measures of involuntary response to disgusting stimuli.

Just as employing self-reports to tap sensitivity to disgust has empirical validation so too does employing physiological measures. In fact, one school of thought, dating back to William James [25], holds that homeostatic physiological activity in response to a stimulus comes first and is subsequently represented in subjective feelings when emotionally-relevant parts of the brain become aware of the peripheral physiological responses. Whether emotions are physiological changes or conscious subjective feelings may be in dispute but the relevance of peripheral physiological activity to emotions is not. This statement is particularly true of the emotion of central concern here. Subsequent to exposure to disgusting stimuli, cardiovascular and gastric (electrogastrogram) readings have been found to correlate with neural activity in disgust centers of the brain. In addition, each type of disgust (core as opposed to violations of the body envelope) has distinctive physiological and neural signatures [11]. Other research finds that changes in skin conductance (a standard measure of autonomic arousal) also co-vary with changes in neural activity in the insula and orbitofrontal cortex, among other areas, suggesting that brain “areas implicated in emotion and attention are differentially involved in generation and representation of peripheral SCR responses” [26].

## Methods

### Ethics statement

The study was approved by the Institutional Review Board at the University of Nebraska – Lincoln and all subjects gave written informed consent prior to participation in the study.

Participants were part of a random sample of individuals contacted in the early summer of 2007 by a professional survey research organization retained to conduct a telephone survey of the population of Lincoln, Nebraska (population 275,000). During the initial phone call, respondents were asked three items in order to identify the extent of their political interest and were asked if they would be willing to come to a lab in the city to participate in an experiment. On the basis of their answers to these items (the goal was to select from this initially random sample a subset composed of an equal mix of individuals inclined to the political right, inclined to the political left, and inclined to avoid politics altogether) and scheduling success, 200 individuals traveled to the lab and completed a survey on their political views and personality traits.

The intention was then to use this larger group that had provided background survey information as a pool from which we could draw smaller groups that would participate in subsequent physiological tests as time and funding became available. Accordingly, later that summer a group of 50 of the larger group's most politically interested participants was called back for an analysis of responses focusing on threat and related topics. Then a year later, in the summer of 2008, we invited 50 more subjects back for a series of tests focusing primarily on reactions to disgusting stimuli so it is this second group that forms the basis for the analysis reported below. The 50 individuals participating in the disgust study analyzed here were randomly selected from those 150 participants in the larger group who had not already returned to the lab for the earlier physiological exercise though, in order to assess the longitudinal stability of physiological measures we did randomly select nine who had participated previously (interestingly, for these nine individuals, the correlation of startle eye blink EMG—the only physiological measure obtained in both sessions—in 2007 and in 2008 was an impressive .93). We make no claim that the group employed in our disgust analysis is representative of the country or even of the city from which it was drawn; indeed, representation is not essential for an exercise such as ours. We can say, however, that the group is not composed of undergraduates and looks reasonably typical with regard to standard demographics: mean age = 41, 55 percent female, mean income $40,000 - $60,000.

Each of the sessions—the initial computer-based survey in 2007 and the disgust-centered physiological exercise in 2008—lasted approximately an hour, including the time required to secure informed consent and to debrief the participants. Individuals were given $50 for each separate visit to the laboratory. Among the survey items were several soliciting opinions on 16 brief issue-prompts, presented in the well-known Wilson-Patterson format [27], in which respondents indicate agreement or disagreement with a word or short phrase (they could also equivocate). The specific issues are listed in Table 1 and cover a range of topics. Most importantly, included in this list is an item on gay marriage and two other items related to sex. It is on these issues and especially the issue of gay marriage that we expect physiological variations in response to disgusting stimuli to have their strongest effect. Consistent with theory and previous findings, we have little reason to expect that physiological responses to disgust would be related to issues involving the economy or national defense and these items are included in Table 1 primarily to provide a contrast with attitudes toward gay marriage.

**Table 1:** Political Orientations and Response to Disgusting Stimuli.

Variable	Skin conductance change (controlling for age, gender, and education)	Skin conductance change (controlling for age, gender, education and self-reported disgust sensitivity)	Self-reported disgust sensitivity (controlling for age, gender, education and skin conductance change)
*Self-Reported Ideology*	.29**	.28*	.11
*Wilson-Patterson Item*			
Gay Marriage	.44**	.45**	.30**
Pre-marital Sex	.28*	.29*	.36**
Abortion Rights	.09	.17	.29*
Free Trade	.07	.06	−.07
Small Govt.	−.12	−.19	−.19
Illegal Immigrants	−.06	−.03	−.00
Military Spending	−.14	−.12	.04
Foreign Aid	.08	−.01	−.16
Police Searches	−.14	−.15	−.11
School Prayer	.05	.09	.01
Gun Control	.10	−.00	−.26
Death Penalty	−.07	−.14	.06
Biblical Truth	.12	.11	.23
Pornography	.10	.11	.05
Tax Cuts	.01	−.00	.08
Welfare Spending	.22	.16	−.10
*5-Pt. Gay Marriage Item*	.37**	.39**	.39**

A single asterisk.

*indicates p<.10, two-tailed.

A double asterisk.

**indicates p<.05, two-tailed.

To measure self-reported response to disgusting stimuli, we followed previous research by administering the 25-item DS-R and then creating an index of only the core and contamination subfactor items. Our central concern, however, is obtaining a physiological, rather than self-reported, measure of response to disgusting stimuli. To construct such a measure we measured skin conductance levels (SCL) while participants viewed a series of 38 images, many of which came from the International Affective Picture System (IAPS) collection, a widely used standard for visual stimuli in psychological studies [28]. This approach of relying on visual images recommends itself in light of findings indicating highly similar neural activity (particularly in the anterior insula) regardless of whether an individual tastes something disgusting, sees something disgusting, or is asked to imagine something disgusting [29].

Subjects were told that they would be viewing a series of images and then each image was presented on a computer screen for 15 seconds and separated from succeeding images by an inter-stimulus interval (ISI) of ten seconds that consisted of a focus point (a large X on an otherwise blank screen). During image and ISI exposure SCL was collected from participants using a pair of Ag/AgCl electrodes. An isotonic contact medium was applied on a 1 cm area using a circular adhesive collar on the distal phalange of the index and middle fingers of the left hand, and skin conductance was transduced using a 0.5 Vrms, 30 Hz sinusoidal excitation signal via an Isolated Bioelectric Amplifer built by James Long Company, Caroga Lake, NY. The signal was digitized at 1 kHz and stored on disk. The sequence of the images was randomized once and then presented to all participants in that same order. Because baseline skin conductance levels differ widely from individual to individual as a function of variations in thickness of skin and numerous other factors, an effective and commonly-employed strategy to control for this variation is to use first differences. Thus, consistent with established practice [30], our key physiological measure is not the absolute skin conductance levels evident during viewing of the identified image but rather the change in mean skin conductance levels from that registered during the previous ISI to that registered during viewing of the stimulus in question. The average change in logged skin conductance precipitated by viewing the three disgusting stimuli thus constitutes our primary measure of physiological response to an image stimulus.

**Figure 1:**
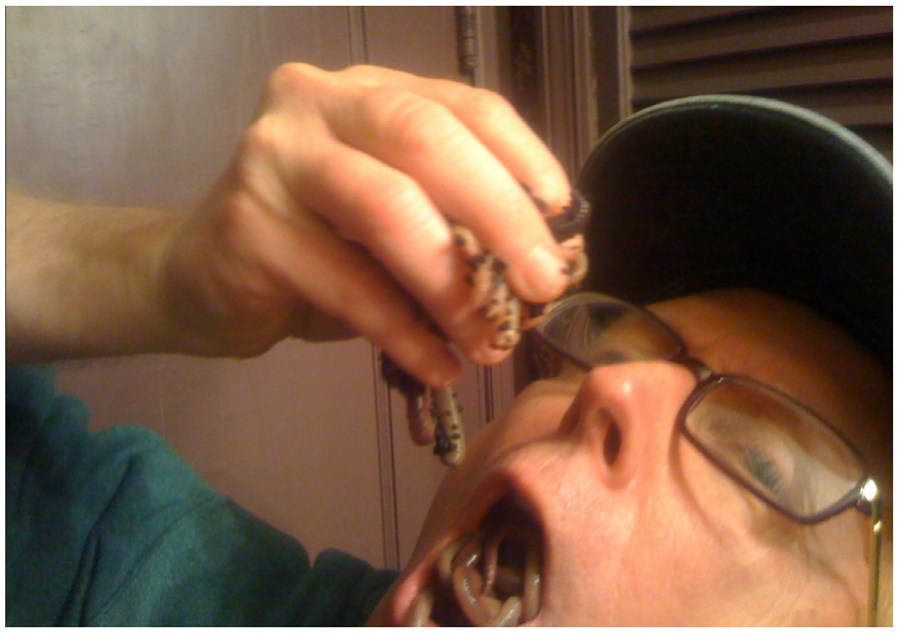
Sample of Type of Image Rated to be Disgusting. This is an image similar to one of the actual image types rated as disgusting by the raters. The actual images used in the study are from the International Affective Picture System (IAPS) collection and cannot be reproduced in a publication. This image is modeled on an image in the IAPS collection of a man eating worms, but the man in this picture is actually one of the authors of this article.

Images were selected to provide variation in emotional valence (positive to negative) and arousal (low to high), and ranged from a positively valanced but minimally arousing image of a bowl of fruit, to a negatively valenced and highly arousing image of a screwdriver poking towards a human eye, to relatively neutral images such as a photograph of a room. All images were independently rated by 126 judges (undergraduates who rated the images for course credit). Judges were asked to rate each image in three ways: (1) valence using a 1–9 scale (“How does this image make you feel?” with response categories ranging from 1 = image evoked happy/positive feelings to 9 = image evoked unhappy/negative feelings), (2) intensity using a 1–9 scale (“How strong is the emotional reaction you feel?” with response categories ranging from 1 = no reaction to 9 = strong reaction), (3) identification of specific emotion evoked by each image. To provide the latter, judges were given a list of 12 emotions (happiness, satisfaction, surprise, anxiety, fear, disgust, grief, anger, sadness, excitement, boredom, amusement) and asked to indicate which emotions were evoked by the image. We deliberately sought variation in emotional specificity, valence, and arousal, in an attempt to empirically isolate specific emotional targets. Of the 38 images viewed by our research subjects, five were identified by our judges as unambiguously negatively valenced, highly arousing and evoking the specific emotion of disgust (defined as at least 80 percent of judges identifying the image as having a mean arousal rating of at least 6.0 on the 1–9 scale, where nine is the most arousing). The five images meeting these criteria were a man in the process of eating a mouthful of writhing worms, a horribly emaciated but alive body, human excrement floating in a toilet, a bloody wound, and an open sore with maggots in it (to illustrate the nature of the images employed, Figure 1 presents an image of a man eating worms). The means for these five images are: 87 percent reporting disgust, 7.31 arousal rating, and a valence rating of 7.59.

The procedures just described allow us to identify and isolate images specifically associated with disgust (as opposed to other negatively valanced emotions such as fear or anxiety), but they do not partition the five key images into the core/contamination/animal reminder disgust categories identified by Haidt and Graham [17]. This is important because theoretically the focus needs to be on core/contamination categories. Of the five images, the bloody wound and the open sore clearly evoke animal reminder disgust as described by Haidt and Graham [17] in that they focus on violations of the bodily envelope. The other three are clearly core/contamination stimuli, focusing as they do on questionable foods, bodily emissions, and a possibly contagious human body, respectively. Consistent with these prima facie, but subjective, observations, factor analysis revealed that responses to three of the five images loaded on a common core/contamination factor but that the other two did not. The two that did not load on a common factor were indeed the animal reminder images. After removing them, we are thus left with three images that are evocative and that fit in the core/contamination disgust categories.

## Results

**Figure 2:**
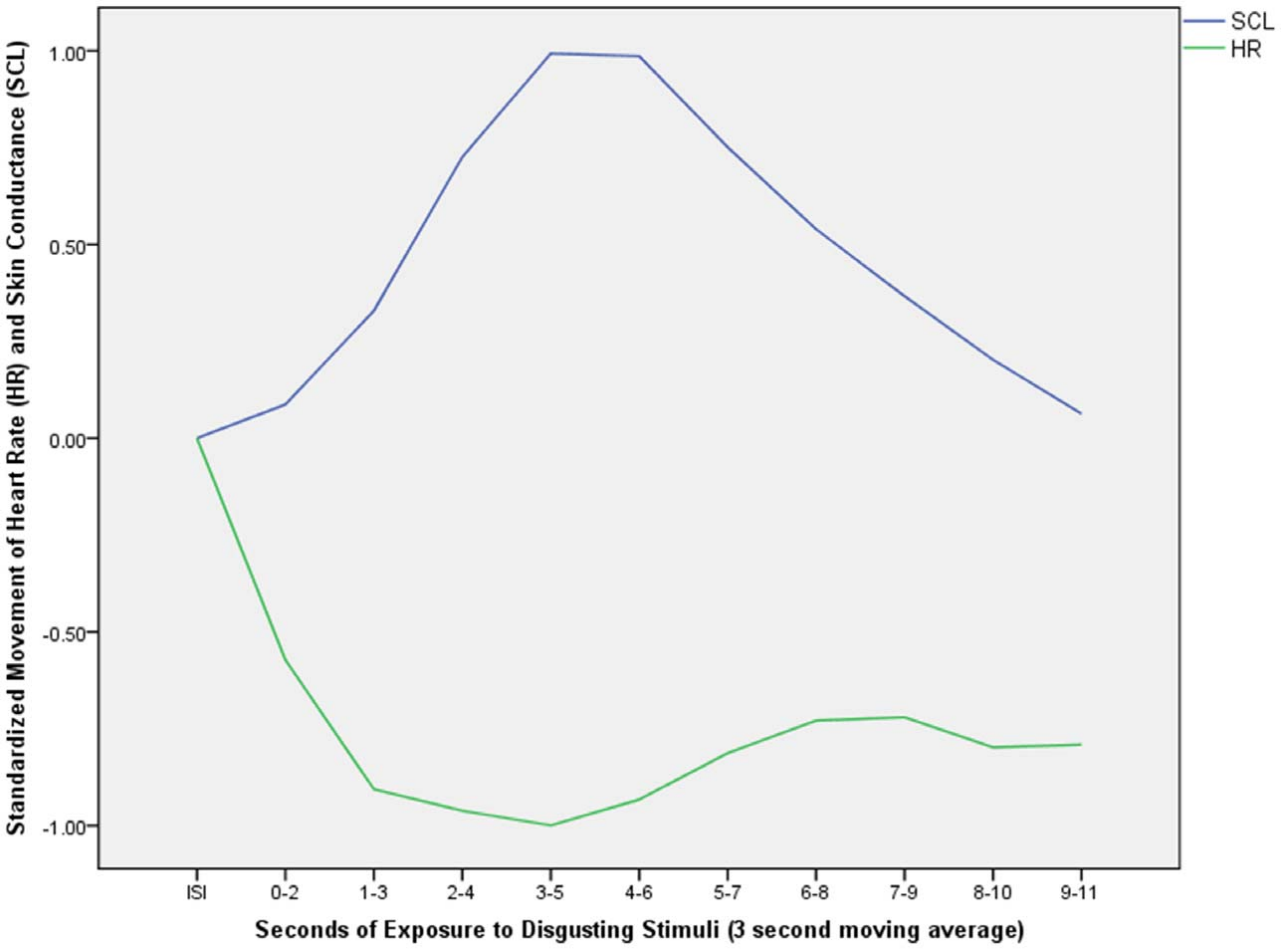
Trends in Skin Conductance and Heart Rate during Exposure to Disgusting Stimuli. The lines represent second by second readings (smoothed into three-second moving averages) of skin conductance levels or SCL (top line) and heart rate or HR (bottom line) during disgust image exposure as a proportion of these same physiological readings during the preceding inter-stimulus interval (ISI). For ease of interpretation, baselines have been standardized to equal 0. Positive numbers indicate an increase in the physiological response during viewing of the disgusting image relative to that existing during viewing of the ISI; negative numbers indicate a decrease.

Before correlating political orientations with skin conductance changes elicited by disgusting stimuli, we first present an overview of the mean physiological trends for all respondents across the first 10 seconds of the showing of the three disgusting images. Even though our interest is in changes in skin conductance, to provide a point of comparison we also include the pattern for heart rate (beats per minute). The lines in Figure 2 represent second by second readings (smoothed into three-second moving averages) of skin conductance levels or SCL (top line) and heart rate or HR (bottom line) during disgust image exposure as a proportion of these same physiological readings during the preceding ISI. For ease of interpretation, baselines have been standardized to equal 0. Positive numbers indicate an increase in the physiological response during viewing of the disgusting image relative to that existing during viewing of the ISI; negative numbers indicate a decrease. Because the units of the two variables in the figure (microsiemens and beats per minute, respectively) are so different, the range of each measure has been standardized to run from 0 to 1. This standardization means the magnitude of movement should not be compared from one measure to the other, even as the figure does provide useful information on the direction, timing, and contours of each measure's movement.

With regard to skin conductance, Figure 2 shows the fairly quick increase and then long, gradual decline that is characteristic of electrodermal response [31]. Also typical is the “triphasic” pattern evident for heart rate in response to disgusting stimuli (fairly sudden and steep decline followed first by a less dramatic increase and then by a flattening out or mild decline) [32]. Whereas threatening stimuli tend to elevate both heart rate and skin conductance, disgusting stimuli depress heart rate but stimulate skin conductance [32], [33]. Thus, Figure 1 is useful in demonstrating that the overall mean physiological patterns observed in our data are consistent with disgust stimulus responses reported in the broader psychophysiology literature.

Responses to disgusting stimuli are thought to be related to gender and perhaps to other demographic factors [10], [22], so in testing for the relationship between political orientations and physiological reactions to disgusting stimuli, we control for the standard variables of age, gender, and education (as reported by the participants in the demographic section of the survey administered to the larger group). “Conservative” positions on the issue items are given higher codings and larger skin conductance increases indicate a greater mean electrodermal response so, in light of the theory and findings just summarized, a positive relationship is hypothesized for gay marriage and, to a lesser extent, for the other sex-related issues but not for the remaining individual political issues.

This predicted pattern is precisely the one appearing in the results. In the first row of the first column of Table 1, in order to provide an overall perspective, we test for the connection, controlling for basic demographic factors, between physiological responses to disgust and self-professed ideological conservatism (are you a liberal, a conservative, or a moderate?). It does indeed appear that self-professed conservatives are somewhat more physiologically responsive to disgusting stimuli, but our primary interest is in the relationship of these physiological responses to specific issue attitudes. Here we see that the correlation of physiological response to disgusting images and opposition to gay marriage is positive, sizable, and statistically significant. In fact, of the 16 Wilson-Patterson items, gay marriage is the only issue that is significantly related (p<.05) to electrodermal response. Tellingly, the only other one that comes close (r = .28; p<.10) is pre-marital sex. As expected, coefficients for the remaining issues are generally small, statistically insignificant, and about as likely to be negatively as positively signed.

The Wilson-Patterson format makes it possible to test a large number of issues in a short amount of survey time but the response set is obviously restrictive. Fortunately, our survey also included five issue items with five-point response options and one of these items also tapped varying levels of support for “a constitutional amendment to ban gay marriages.” The relevant coefficient is the last one in column 1 of Table 1 and it shows that the relationship between attitudes on gay marriage and changes in skin conductance while viewing disgusting stimuli is once again sizable, positive, and statistically significant. In fact, the gay marriage item is the only five-point issue item of which this can be said (the others asked about attitudes toward taxes, energy policy, healthcare, and abortion rights). It appears that those individuals who have the strongest physiological responses to an array of disgusting stimuli (none of which directly relates to sexuality or homosexuality) also tend to be the individuals who oppose gay marriage. In some respects, it is quite remarkable that involuntary physiological responses to non-political stimuli exert such an effect on two different measures of the specific political attitude hypothesized to be affected and not on any others.

One of the advantages of our data set is that we have measures of both physiological responses to disgusting stimuli and self-reported sensitivity to disgusting concepts, thus making it possible to test for the independent effects of each. Neither the self-report measures nor the physiology measure should be taken to constitute the “real” indicator of response to disgust; rather, these are two valid but very different approaches to measurement and, even though our emphasis in this article is on a physiological variable, its value can best be identified by simultaneously taking into consideration the established role of self-reports. Physiological and self-report measures may very well each pick up distinct elements of response to disgusting stimuli and therefore may be independently useful.

Analysis of this matter begins with a surprise: our measure of physiological response to disgusting stimuli is uncorrelated with self-reported disgust sensitivity. Neither the recommended 5-item contamination subscale (p = .21) nor the full 25-item DS-R (p = .32) is close to being statistically significant when correlated with changes in skin conductance while viewing disgusting stimuli. One possible reason for the absence of a bivariate relationship is the potentially confounding effects of gender. Physiological and other differences between males and females may need to be controlled in order for a relationship between physiological response and self-reported disgust sensitivity to appear. Interestingly, while the data do indeed suggest important differences between males and females, these differences do not seem to be physiological and they do not seem to be the reason for the absence of a correlation.

**Figure 3:**
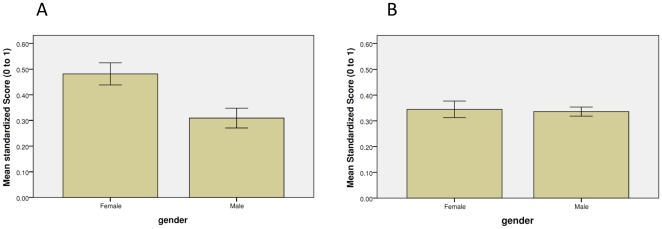
Gender and Response to Disgusting Stimuli. Panel A presents the mean scores for the self-report disgust scale and panel B presents the mean scores for skin conductance response. The range of both self-reported and physiological disgust sensitivity has been standardized to run from 0 to 1. Mean gender differences occur for self-reported disgust sensitivity (t = 2.85, p<.01, two-tailed test) but not for physiological disgust sensitivity (t = .22, p = .82, two-tailed test).

As indicated in Figure 3, where the range of both physiological and self-reported disgust sensitivity has been standardized to run from 0 to 1, mean gender differences occur for self-reported disgust sensitivity (p<.05) but not for physiological disgust sensitivity (p = .82). One possible explanation of these results is that females claim to be more disgust sensitive because they feel societal pressure to project sensitivity just as males report being less disgust sensitive because they feel societal pressure to project toughness. Whether or not this interpretation is correct, these findings showing gender differences for self-reported but not physiological response fit with previous studies [34], [35]. Thus, existing scholarship (and folk wisdom) holding that “women are more disgust sensitive than men,” [10], [22] should be changed to women ***report*** being more disgust sensitive than men.

Though the gender differences in self-report are substantial, they do not account for the lack of overall correlation between reported disgust sensitivity and changes in skin conductance when viewing disgusting stimuli. A partial correlation of skin conductance changes and reported disgust sensitivity controlling for the effects of gender still does not produce a statistically significant relationship (p = .23) and in a more fully specified regression format, interactions of gender and each measure of response to disgust were not significantly related to the other measure of response to disgust.

The real issue, however, is the effects of physiological responses and self-reports on political attitudes and a reasonably straightforward way of testing for possible connections is to partial the effects of each while controlling for the other. This is what we do in the final two columns of Table 1. In the middle column, we add self-reported disgust sensitivity (the contamination subscale) to the previously introduced control variables to see if changes in skin conductance still have an independent effect on political orientations. Then in the final column we reverse the analysis such that skin conductance changes are added as a control variable, with self-reported disgust sensitivity becoming the target variable, thereby highlighting any potential independent effects of self-reported disgust sensitivity.

We find that even though self-reported disgust sensitivity and skin conductance change when viewing disgusting images are not significantly related to each other, they are both independently and strongly related to attitudes toward gay marriage. This statement applies to both the Wilson-Patterson item on gay marriage and the separate five-point item. Both measures also appear to have independent effects on attitudes toward premarital sex. Effects on the other specific issues, as expected, are weak across the board.

Since we are not making claims about causal order, to this point we have relied upon partial correlations, but regression analysis makes it possible to highlight the independent contributions of skin conductance and self reports in accounting for variations in attitudes toward gay marriage. We use the five-point rather than Wilson-Patterson gay marriage item in order to minimize violations of the assumptions of OLS regression and we also include the same three control variables as before. The results are as follows:

oppose

gay mar. = .03(age)+.70(gender)−.14(educ.)+.89**(self-report)+38.86**(skin cond.).02)(.44)(.16)(.32)(12.9)

N = 46

R^2^ = .27

Adj R^2^ = .18

F = 3.03**

Unstandardized coefficients (standard errors) reported

* = (p<.10) ** = (p<.05).

Here we see that solid independent effects on gay marriage attitudes are registered for both skin conductance changes and self-reported disgust sensitivity. Both have large, positive, statistically significant effects while the demographic control variables do not, though there are statistically insignificant tendencies for older, less educated males to be opposed to gay marriage. The most important conclusion drawn from these results is that an improved prediction of individuals' attitudes toward gay marriage is made possible by knowing both the extent to which they perceive themselves to be disgust sensitive and the degree to which their skin conductance increases when they are exposed to disgusting images. On the basis of these findings, it would seem that self-reports and physiological measures each have an important independent effect on attitudes toward gay marriage. Even though they are not empirically related to each other, both measures improve specification of the model.

The effect sizes reported here should be kept in perspective. On its own, realistic degrees of change in neither skin conductance nor self-reported disgust sensitivity will create a die-hard supporter or confirmed opponent of gay marriage. The dependent variable in the regression equation runs from 1 (strongly oppose a Constitutional amendment banning gay marriage) to 5 (strongly support an amendment banning gay marriage) and the contamination subscale is a mean of responses to five, 5-item questions, and so has a theoretical range running from 1 to 5 and an actual range in our sample from 1 to 4.2. Given the pertinent coefficient in the above equation, this means a one point increase in the contamination disgust sensitivity scale is predicted to shift an individual .89 on the five point support for gay marriage scale. The measurement metric for skin conductance is more difficult to interpret since it has been logged and first differenced and since most people are not familiar with microsiemens as a unit of measurement. Perhaps the best we can do is to say that, with other variables in the equation held at their means, a one standard deviation increase in mean skin conductance change when viewing the disgusting images would be predicted to increase opposition to gay marriage by about .39 standard deviations. As the standard deviation for the dependent variable is 1.55, a 1 standard deviation increase in skin conductance would translate into an increase in support for an amendment banning gay marriage of roughly .59 points on a five-point scale.

Skin conductance is well known to increase in response to a wide variety of stimuli [33] so there is the possibility that the connection we are proposing between skin conductance increases and political orientations could apply to non-disgusting images as well. Note that such a finding would be equally important for the connection of physiology and politics but it would suggest a different interpretation than we have offered here. To check for this possibility, we returned to the image rating data provided by our judges in order to select images that are negatively valanced, arousing, (6 or above on the 1–9 scale) but evoke an emotional response that was not disgust. The three images that best filled this role, according to our judges, were a house on fire, a large shark swimming ominously close to a kayaker, and an angry dog. These three images had an average valence rating of 6.71 (strongly negative), and an arousal rating of 6.25. On average only 6 percent of respondents judged these images to evoke disgust, while 67 percent judged them to evoke fear (the emotion next most commonly identified with these images was anxiety, with 50 percent of the raters selecting this descriptor). In short, these images seem to evoke negatively valenced (but non-disgust) emotional responses. Accordingly, they should provide a useful initial test of whether attitudes toward gay marriage are predicted by physiological reactivity to any negative images or whether there is something special about disgusting images.

**Figure 4:**
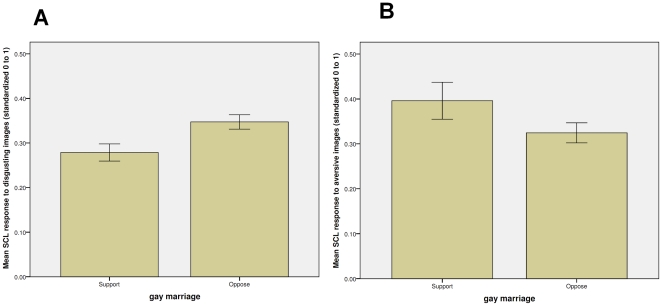
Physiological Response to Disgusting and Other Aversive Stimuli and Attitudes on Gay Marriage. The bars represent the proportion of change from the last second of the inter-stimulus interval (ISI) to the highest skin conductance level (SCL) reading during first six seconds of viewing the stimulus in question. Scores are standardized 0 to 1. The number of subjects is 24 for the ‘support gay marriage’ group and 23 for the ‘oppose gay marriage’ group. Panel A presents the scores for disgust images while panel B presents the results for the aversive non-disgust images. Despite the small number of subjects, skin conductance changes occasioned by the disgusting images distinguish supporters and opponents of gay marriage (t = 3.10, p<.01, two-tailed test). In contrast, skin conductance changes occasioned by the aversive but not disgusting images are quite similar for supporters and opponents of gay marriage (t = .24, p = .80, two-tailed test).

We present the results graphically in Figure 4, taking advantage of the essentially dichotomous nature of the Wilson-Patterson format (only 3 respondents claimed to be undecided about gay marriage and they are excluded from the figure) to contrast the different patterns depending upon whether the images were disgusting or were aversive but not disgusting. The bars represent the change from the last second of the ISI to the highest SCL reading during viewing of the stimulus in question. Significance levels are slightly reduced as a result of the dichotomized dependent variable and the loss of cases but the visual representation is helpful. As can be seen, skin conductance changes occasioned by the disgusting images distinguish supporters and opponents of gay marriage whereas skin conductance changes occasioned by the aversive but not disgusting images are quite similar for supporters and opponents of gay marriage. Also of note is the fact that, in our sample at least, skin conductance changes in response to disgusting stimuli do not correlate highly with skin conductance changes in response to other aversive stimuli (p = .489), suggesting physiological reactivity varies depending upon the stimulus type in question [33], a finding consistent with evidence that disgust and disease avoidance activate different neural pathways from threat (self-protection) and other responses to aversive situations [13]. In any event, a thorough investigation of the match between responses to discrete categories of stimuli and stances on particular political issues awaits studies with larger Ns and broader ranges of stimuli, but our initial investigation points to the conclusion that attitudes toward gay marriage (and perhaps other sex-related issues) have a special connection to disgust.

## Discussion

Mounting evidence points to the relevance of subconscious factors in broad social, decision-making situations [36]–[38] and in specifically political decision-making situations [39]–[48]. The established role of such factors opens the door for the possible involvement of biological variables, including hormone and neurotransmitter levels [49]–[52] and neural traits and patterns [7], [53]–[56]. This stream of research is not entirely consistent with the general thrust in political science research which holds that political orientations come from “direct involvement with the raw materials of politics” and are shielded from extraneous influences [57], [58] and as such has the potential to alter knowledge of the source of political orientations.

Still, it is important to recognize that our results are only correlational. Accordingly, we cannot be certain whether reactions to disgusting stimuli—either self reported or physiological—precede, follow, or are coterminous with political orientations, though we tend to agree with Inbar, Pizarro, and Bloom that it seems “unlikely political attitudes would shift a person's general emotional dispositions, particularly when it comes to disgust, a basic emotion that emerges long before individuals form political attitudes” [10]. It is more likely that these “general emotional predispositions” come before or emerge simultaneously with political orientations. The correlational nature of the findings also means that, even though our results suggest a realistic mechanism by which genes could ultimately link to political orientations through physiological systems, they certainly do not prove that this linkage exists. Physiological responses such as electrodermal activity are quite consistent over time [59] but are the result of both genetics and important experiences. Regardless, debates about causal order miss the larger point. The central implication of our research is that, whether the relevant raw material of political attitudes is entirely environmental or partially innate, these attitudes sometimes become biologically instantiated in involuntary physiological responses to facets of life far detached from the political issues of the day. Moreover, our results indicate that this biological instantiation makes a difference even when controlling for the effects of survey self-reports. To put it differently, the proper interpretation of the findings reported here is not that biology causes politics or that politics causes biology but that certain political orientations at some unspecified point become housed in our biology, with meaningful political consequences.

Acceptance of the role of involuntary physiological responses is not easy for many people. Most are proud of their political orientations, believe them to be rational responses to the world around them, and are reluctant to concede that subconscious predispositions play any role in shaping them. Indeed, since the predispositions are in part subconscious, people are by definition unaware of them. Still, if recognition of the relevance to politics of involuntary physiology became more widespread, it could diminish the frustration generated by the apparent illogical intransigence of political opponents (biologically instantiated orientations are certainly changeable but likely are more difficult to change than orientations lacking such instantiation). This recognition could in turn diminish political hostility. After all, if political differences are traceable in part to the fact that people vary in the way they physically experience the world, certitude that any particular worldview is objectively correct may abate, lessening the hubris that fuels political conflict.
